# Supplementary material for: Causal interplay between lactose intolerance and gut microbiota: a combined bidirectional Mendelian randomization and in vivo validation study
Source: Front Nutr. 2026 Jun 1;13:1803337. doi: 10.3389/fnut.2026.1803337 (PMC13265576; doi:10.3389/fnut.2026.1803337)
Supplement: Supplementary file 1 [file Data_Sheet_1.zip › supplementary materials/Forward/leave one out cross validation/ebi-a-GCST90027709.finngen_R12_E4_LACTONAS.pdf]

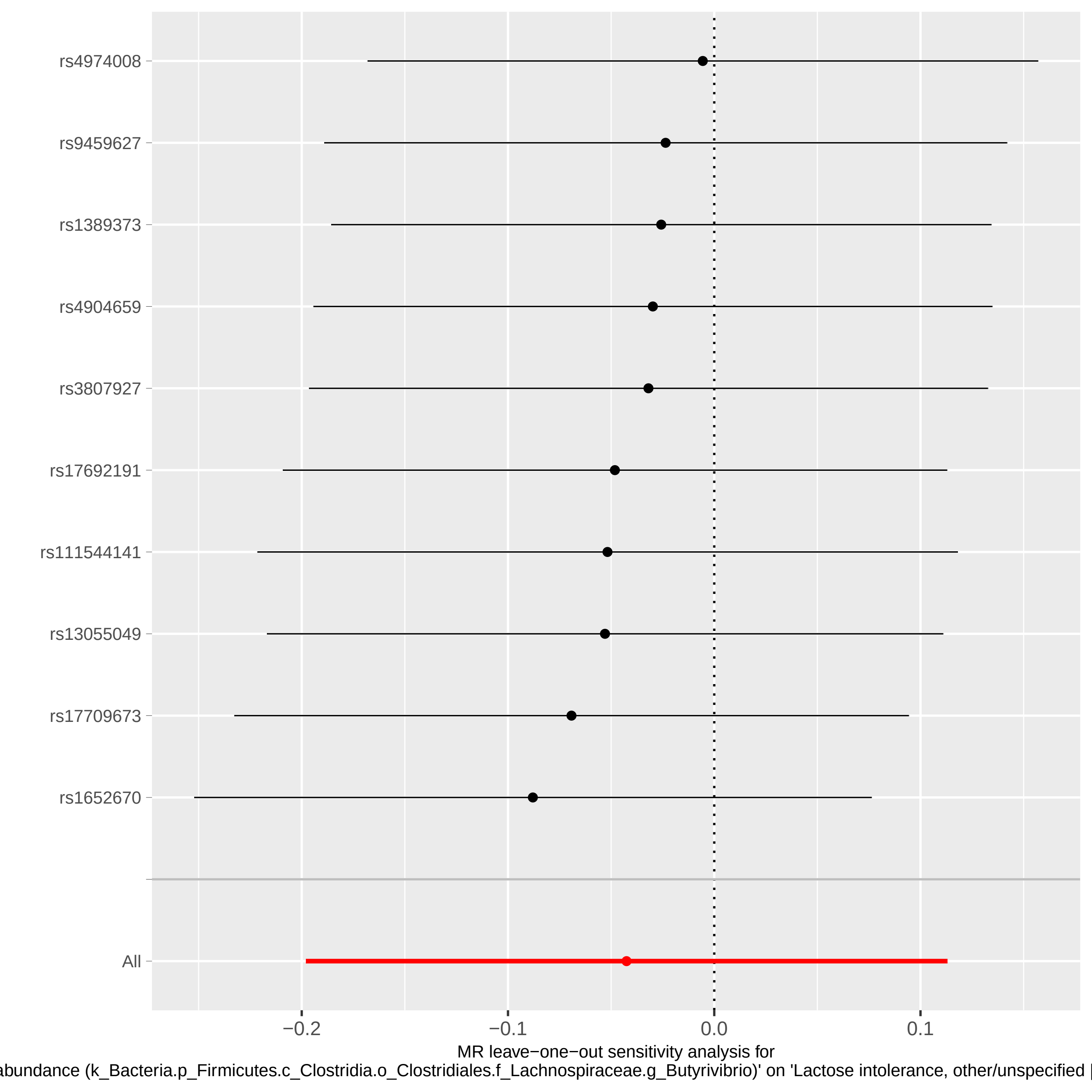
rs4974008
rs9459627
rs1389373
rs4904659
rs3807927
rs17692191
rs111544141
rs13055049
rs17709673
rs1652670
All
−0.2
−0.1
0.0
0.1
MR leave−one−out sensitivity analysis for
abundance (k_Bacteria.p_Firmicutes.c_Clostridia.o_Clostridiales.f_Lachnospiraceae.g_Butyrivibrio)' on 'Lactose intolerance, other/unspecified
